# Chemomics-Integrated Proteomics Analysis of Jie-Geng-Tang to Ameliorate Lipopolysaccharide-Induced Acute Lung Injury in Mice

**DOI:** 10.1155/2016/7379146

**Published:** 2016-08-07

**Authors:** Jin Tao, Yan Nie, Yuanyuan Hou, Xiaoyao Ma, Guoyu Ding, Jie Gao, Min Jiang, Gang Bai

**Affiliations:** State Key Laboratory of Medicinal Chemical Biology, College of Pharmacy and Tianjin Key Laboratory of Molecular Drug Research, Nankai University, Haihe Education Park, 38 Tongyan Road, Tianjin 300353, China

## Abstract

Jie-Geng-Tang (JGT), a classic and famous traditional Chinese medicine (TCM) prescription composed of* Platycodon grandiflorum* (Jacq.) A. DC. (PG) and* Glycyrrhiza uralensis* Fisch. (GU), is well known for “clearing heat and relieving toxicity” and its ability to “diffuse the lung and relieve sore throat.” However, the mechanism underlying its action remains unclear. In this study, potential anti-inflammatory ingredients were screened and submitted to PharmMapper and the KEGG bioinformatics website to predict the target proteins and related pathways, respectively. Differentially expressed candidate proteins from acute lung injury (ALI) mice treated with JGT were identified by isobaric tags for relative and absolute quantitation (iTRAQ) and LC Triple-TOF. Eleven potential anti-inflammatory ingredients were found, including the derivatives of glycyrrhizic acid, licorice-saponin, liquiritin, and platycodigenin. A total of sixty-seven differentially expressed proteins were confirmed after JGT treatment with four therapeutic functions, including immunoregulation, anti-inflammation, ribosome, and muscle contraction. PG and GU comediate PI3K/Akt signal pathway inhibition of NF-*κ*B, VCAM1, and ICAM1 release which primarily act on PI3K, PDK1, AKT, and GSK3*β*. GU markedly inhibits the ERK/MAPK signaling pathways and primarily acts on LCK, RAS, and MEK. A network was constructed using bioactive ingredients, targets, and pathways to determine the mechanism underlying JGT treatment of ALI.

## 1. Introduction

Acute lung injury (ALI) is characterized by hypoxemic respiratory insufficiency from noncardiogenic pulmonary edema caused by increased pulmonary vascular permeability. ALI and its more severe form (acute respiratory distress syndrome) comprise a uniform response of the lung to infectious, inflammatory, or chemical insults and therefore are commonly associated with systemic illness [1]. Inflammatory stimuli from microbial pathogens, such as lipopolysaccharide (LPS), are recognized for their ability to induce pulmonary inflammation, and experimental administration of LPS has been used to induce pulmonary inflammation in animal ALI models [2, 3].

An increasing amount of evidence has demonstrated that LPS can trigger the most potent microbial initiators of inflammatory responses by activating numerous inflammatory cells to release proinflammatory factors, such as TNF-*α* and IL-8 [4, 5]. Several candidate therapeutic strategies, such as fluid management, surfactants, glucocorticoids, and stem cells, have been applied to treat ALI in the last decade. However, the mortality resulting from these conditions remains high. Thus, seeking effective and safe medicines to cure ALI is imperative [6].

Zhang Zhongjing square Jie-Geng-Tang (JGT) is composed of* Platycodon grandiflorum* (Jacq.) A. DC. (PG) and* Glycyrrhiza uralensis* Fisch. (GU) that are boiled in a 1 : 2 ratio. GU is used for “clearing heat” and “relieving toxicity,” whereas PG is well known for “diffusing the lung” and “relieving sore throat” [7]. Several studies have demonstrated that some bioactive compounds from JGT, such as glycyrrhizic acid and platycodin D, attenuate ALI* via* NF-*κ*B and cytokines. Nonetheless, the synergistic anti-inflammatory mechanism of PG and GU is not clear [8, 9].

Network analysis technology is widely used to study the mechanisms underlying the actions of traditional Chinese medicine (TCM), including chemome, genome, and proteomics approaches. These techniques provide an opportunity to clarify the relationships among herbs, compounds, targets, and diseases [10]. Sun et al. studied the mechanisms of the famous TCM formula Yin-Chen-Hao-Tang using proteomics. Two-dimensional gel electrophoresis-based proteomics and MALDI-TOF/TOF-MS were performed on animals treated with the formula to identify target proteins. Differentially expressed proteins are related to functional pathways. Thus, network analysis technology helped elucidate the molecular mechanisms of TCM [11].

In the current study, chemomics-integrated proteomics was introduced to explore the bioactive compounds and the potential anti-inflammatory mechanism of JGT for the treatment of LPS-induced ALI in mice. To screen the bioactive components in this remedy, we developed a dual-luciferase reporter assay-guided UPLC-Q/TOF system for NF-*κ*B inhibitor screening. The TNF-*α* and IL-8 expression levels were utilized to evaluate the JGT treatment efficacy. Differentially expressed proteins were confirmed based on iTRAQ and LC Triple-TOF-based proteomic technology. AutoDock analysis was applied to predict the targets of the compounds in the pathway. Subsequently, network pharmacology based on the bioactive ingredients and the prediction of targets and pathways was used to construct the schematic of the anti-inflammatory mechanism.

## 2. Materials and Methods

### 2.1. Reagents and Chemicals

LC/MS grade acetonitrile was obtained from Merck (Darmstadt, Germany). LC grade formic acid was obtained from Acros Organics (Geel, Belgium), and HPLC water was prepared using the Milli-Q system (Millipore, Bedford, MA, USA). Mouse TNF-*α* and IL-8 ELISA kits were obtained from Pierce/Endogen Co. (Rockford, IL, USA). The reporter plasmids pGL4.32 and pRL-TK were purchased from Promega (WI, USA). Human TNF-*α* was obtained from PeproTech (Rocky Hill, NJ, USA). LPS and dexamethasone (Dex) were purchased from Sigma Chemical Co. (St. Louis, MO, USA). The Lipofectamine 2000 transfection reagent was obtained from Invitrogen (Carlsbad, CA, USA). All cell culture reagents were purchased from Gibco BRL Life Technologies (Rockville, MD, USA).

### 2.2. JGT Extract Sample Preparation

PG (batch number 20100121) and GU (batch number 20090401) were purchased from Qirui Pharmaceutical Company in Anguo and identified by Professor Tiejun Zhang from the Tianjin Institute of Pharmaceutical Research. Based on the “Treatise on Exogenous Febrile Diseases,” sixty grams of PG and GU was boiled at a 1 : 2 ratio in 2 L of water until a 1.3 L solution was obtained. The solution was filtered, evaporated to dryness (13.8977 g), and stored at 4°C. The concentrate was dissolved in water to a 10 mg/mL concentration and filtered through a 0.22 *μ*m filter prior to the UPLC/MS analysis.

### 2.3. UPLC/Q-TOF-MS Analysis

A Waters Acquity UPLC System (Waters Co., Milford, MA, USA) equipped with a photodiode array detector was used. UV detection was achieved in the range of 190 to 400 nm. An Acquity BEH C18 column (2.1 × 100 mm, 1.7 *μ*m; Waters Co.) was used for the separation. Gradient elution of acetonitrile (A) and 0.1% formic acid (B) was performed as follows: 0 min, 2% A; 22 min, 60% A; 23 min, 100% A. The flow rate was 0.30 mL/min and the column temperature was maintained at 30°C. The injection volume of the test sample was 2 *μ*L. Subsequently, the UPLC fractions were partially collected in a 96-well deep-well plate every 0.5 min and then evaporated in a vacuum drying oven at 60°C. The residues were dissolved in 100 *μ*L of cell culture medium for the bioactivity assay.

Mass spectrometry was performed on a Waters Q-TOF Premier instrument with an electrospray ionization system (Waters MS Technologies, Manchester, UK). The ESI-MS spectra were acquired in both the negative and the positive ion modes. The capillary voltage was set to 2.5 kV for the negative mode and 3.0 kV for the positive mode, and the sample cone voltage was set to 30 V. The nebulization gas was set to 600 L/h at a temperature of 350°C, the cone gas was set to 50 L/h, and the source temperature was 110°C. The Q-TOF Premier acquisition rate was 0.1 s with a 0.02 s interscan delay. The instrument was operated with the first resolving quadrupole in a wide pass mode (50–2,500 Da).

### 2.4. Cell Culture, Transfection, and Dual-Luciferase Assay for NF-*κ*B Inhibitors

Human embryonic kidney (HEK) 293 cells obtained from the American Type Culture Collection (Rockville, MD, USA) were grown in Dulbecco's modified Eagle's medium (Gibco BRL) containing 10% fetal bovine serum (Gibco BRL), 100 U/mL of penicillin, and 0.1 mg/mL of streptomycin. The cells were grown to confluence in 96-well plates at 37°C in a humidified incubator with 5% CO_2_.

HEK 293 cells were cotransfected with the NF-*κ*B luciferase reporter plasmid pGL4.32 (100 ng per well) and the Renilla luciferase reporter vector plasmid pRL-TK (9.6 ng per well). The transfection was performed for 24 h using Lipofectamine 2000 according to the manufacturer's instructions. After transfection, the cells were pretreated with the UPLC fractions for 1 h prior to TNF-*α* stimulation (5 ng/mL) for 6 h.

After the samples were stimulated, the HEK 293 cells were lysed and assayed for luciferase activity using a dual-luciferase reporter assay system according to the manufacturer's instructions. The relative luciferase activity was obtained by normalizing the firefly luciferase activity against the activity of the internal Renilla luciferase control (Modulus*™*, Turner BioSystems, USA).

### 2.5. Experimental Animals and LPS-Induced ALI

Male BALB/c mice weighing 18–22 g were purchased from Vital River Company (Beijing, China) and housed in a unidirectional airflow room under controlled temperature (20–24°C), relative humidity (40–60%), and a 12 h light/dark cycle. Filtered tap water and commercial rodent chow were available* ad libitum*. The animals were allowed 3 days to adjust to the environment prior to the experiments and were fasted for 12 h prior to the start of the experiment.

Thirty-six mice were randomly divided into six groups, including an uninfected control (Con) group and five LPS-infected groups as follows: model (Mod) group, Dex group (5 mg/kg, served as positive control), and the JGT groups (0.45 g/kg, 1.35 g/kg, or 4.05 g/kg). Dex and the three JGT doses were orally administered for one week prior to the LPS challenge, whereas the mice from the Con and Mod groups received an equal volume of 0.9% saline instead of the drugs. To induce ALI, the mice were intranasally given 0.5 mg/kg of LPS. The mice were killed 24 h after the LPS challenge. Bronchoalveolar lavage fluid (BALF) was collected three times through a tracheal cannula with 0.5 mL of autoclaved phosphate buffered saline (pH 7.2). Lung tissue samples were harvested at the same time.

### 2.6. Measurements of Inflammatory Mediators

Cytokines (TNF-*α*) and chemokines (IL-8) were measured in the BALF. The BALF were centrifuged at 1000 ×g for 10 min at 4°C, and the TNF-*α* and IL-8 levels in the supernatants were evaluated by ELISA kits in accordance with the manufacturer's protocol.

### 2.7. Histopathological Evaluation of the Lung Tissues

A histopathological examination (HE) was performed on mice not subjected to BALF collection. The lungs were fixed with 10% buffered formalin, embedded in paraffin, and sliced. After hematoxylin and eosin staining, pathological changes in the lung tissues were observed under a light microscope (Olympus CKX41, Japan). The degree of microscopic injury was evaluated based on the neutrophil infiltration and alveolar density using MATLAB software system (MathWorks, 2013b, USA), which could quantify the severity of inflammation after converting the picture to a grayscale image. An edge detection algorithm for grayscale images was applied to average each image into 100 rectangular units. The intensity of each rectangle of the lung parenchyma was cumulative and was divided by the corresponding pixels. The intensity of each part was normalized and emerged as a percentage of the gray value. The alveolar density, which is an index used to depict the severity of lung injury, was calculated for four sections of the overall integrated image from each group.

### 2.8. Forecast Analysis of Potential Functional Targets and Pathways

The PharmMapper database (http://lilab.ecust.edu.cn/pharmmapper/) was used to transform the compounds with anti-inflammatory activity via reverse molecular docking analysis to predict the primary targets. Then, the predicted targets were annotated by the KEGG database (http://www.genome.jp/kegg/) to obtain the related pathways. Identical interactions in the subnetwork and network may have overlapped. The chemical compounds-targets-pathways were annotated in the anti-inflammatory disease subnetwork using Cytoscape (Ver. 2.6.0) to achieve the JGT anti-inflammatory network.

### 2.9. Sample Preparation and Labeling for iTRAQ Analysis

Lung samples from three groups (Con, Mod, and JGT-H) (*n* = 3) were chosen for iTRAQ analysis by the Beijing Genomics Institute in Shenzhen (BGI Tech). The iTRAQ experiment was performed according to the protocol Amine-Modifying Labeling Reagents for Multiplexed Relative and Absolute Protein Quantitation. Briefly, each sample was reduced, alkylated, digested, and labeled with the iTRAQ reagents. After a 2-hour incubation period, the labeled samples were combined and dried prior to strong-cation exchange fractionation. Finally, the LC-ESI-MS/MS analysis based on Triple-TOF 5600 was performed to obtain raw data.

### 2.10. iTRAQ Data Analysis and Function Method Description

The raw data files acquired from the Orbitrap were converted into MGF files using Proteome. Protein identification was performed using the Mascot search engine (Matrix Science, London, UK; version 2.3.02). A protein that contained at least two unique peptides was required for the protein quantitation. The quantitative protein ratios were weighed and normalized by the median ratio in Mascot. The screened proteomes of the three samples were compared in pairs using a pairwise comparison, including Con* versus* Mod, Con* versus* JGT, and Mod* versus* JGT. The former comparison was used as the control and the latter comparisons were considered the treatments. Functional annotations of the proteins were conducted using the Blast2GO program against the nonredundant protein database (nr; NCBI). The KEGG database and the COG database (http://www.ncbi.nlm.nih.gov/COG/) were used to classify and group the identified proteins. The protein interactions were visualized using String 9.1 (http://string-db.org/).

### 2.11. Docking with AutoDock and Free Energy Calculations

AutoDock 4.0 was used to screen the candidate molecules obtained from JGT against target selectivity. The crystal structures of the chosen targets were obtained from the Protein Data Bank (PDB ID: 2UZI, 4ACD, 3EQD, 3OCS, 4KZC, 3KMM, 2R7B, and 3QKK) and optimized using SYBYL X2.0. The target structures were preprocessed and a grid box was generated prior to docking. At this stage, the target structures were prepared by deleting all water molecules and adding all hydrogen atoms. Gasteiger charges were used for the docking. Then, PDBQT files of the targets and ligands were prepared using AutoDock Tools.

The centers of the grids were placed onto the center of ligand mass. A genetic algorithm (GA) was used to simulate ligand-receptor binding. The number of GA runs was 30. The step size parameters of quaternion and torsion were set to 30. A total of 30 independent runs were performed for each compound. The docking parameters were assigned following the strategy proposed in the parameter test section; default values were used for all other parameters.

The free energy of binding calculation was performed using MM/PBSA. The average free energy of the complex, receptor, or ligand was composed of the mechanical energy, energy of solvation, and entropic energy of the system over the trajectory. The single trajectory approach was applied to estimate the energies. This approach extracted the thermodynamic data from a single trajectory of the protein-ligand complex. Because significant binding occurred between the molecules and targets, this error calculation could reduce the effect of incomplete sampling.

### 2.12. Statistical Analysis

Statistical analysis was performed using the SPSS software and the data were presented as the standard error of the mean. Significance was determined by one-way analysis of variance followed by Tukey's multiple comparison test. Significant differences between the means were determined using Student's *t*-test. Statistical significance was set at *p* < 0.05.

## 3. Results

### 3.1. Identification of NF-*κ*B Inhibitors in the JGT Extract by Bioactivity-Based UPLC/Q-TOF

**Figure 1:**
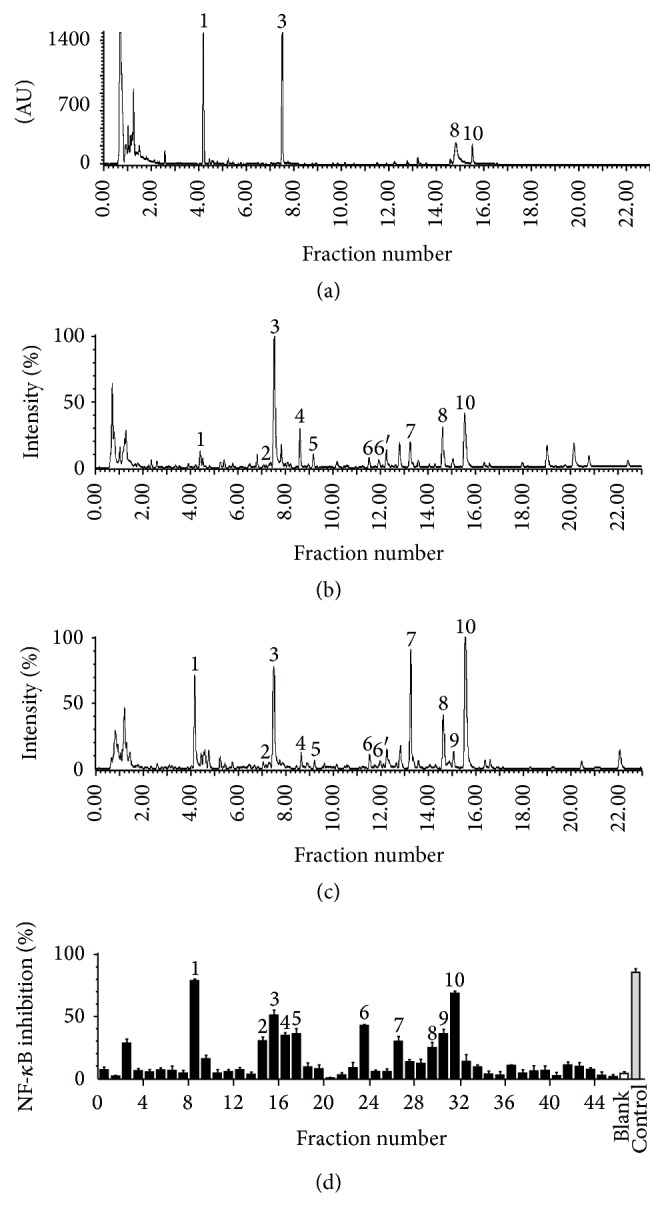
UPLC/Q-TOF-MS and dual-bioactivity analysis of the JGT extract. (a) UPLC/UV chromatograms of the JGT extract. (b and c) TIC chromatograms in positive ESI mode and negative ESI mode, respectively. (d) Bioactivity chromatograms obtained* via* the dual-luciferase reporter assay system for NF-*κ*B inhibition. The peak numbers are consistent with those reported in Table 1.

The optimal UPLC-Q/TOF conditions were applied for the analysis of the JGT extract. The UV (Figure 1(a)) and total ion current chromatograms in the positive and negative ESI modes (Figures 1(b) and 1(c)) are shown in Figure 1. To identify the effective components in JGT, the extract components were separated by UPLC. After UPLC/diode array detection analysis, 90% of the column effluent was collected in 0.5 min fractions for the NF-*κ*B bioactivity assay using the dual-luciferase reporter assay system. Ten fractions showed potential NF-*κ*B inhibition (Figure 1(d)) and eleven anti-inflammatory compounds were identified.

**Figure 2:**
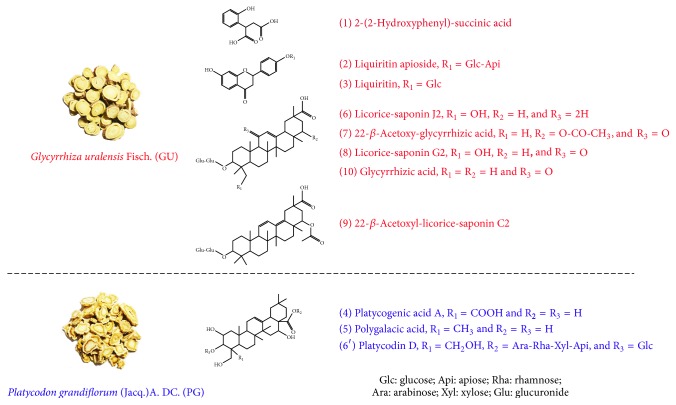
Chemical structures of the bioactive compounds in JGT.

Among the potential active ingredients, eight compounds belonged to GU and three belonged to PG. GU primarily contained polyphenolic compounds (1–3) and pentacyclic triterpenoids (6–10), whereas PG contained terpenes (4, 5, and 6′) [12–14]. The detailed results of the identified compounds and the MS and MS/MS information are shown in Table 1 and the structures of the anti-inflammatory components are shown in Figure 2.

### 3.2. Histopathological Examination

**Figure 3:**
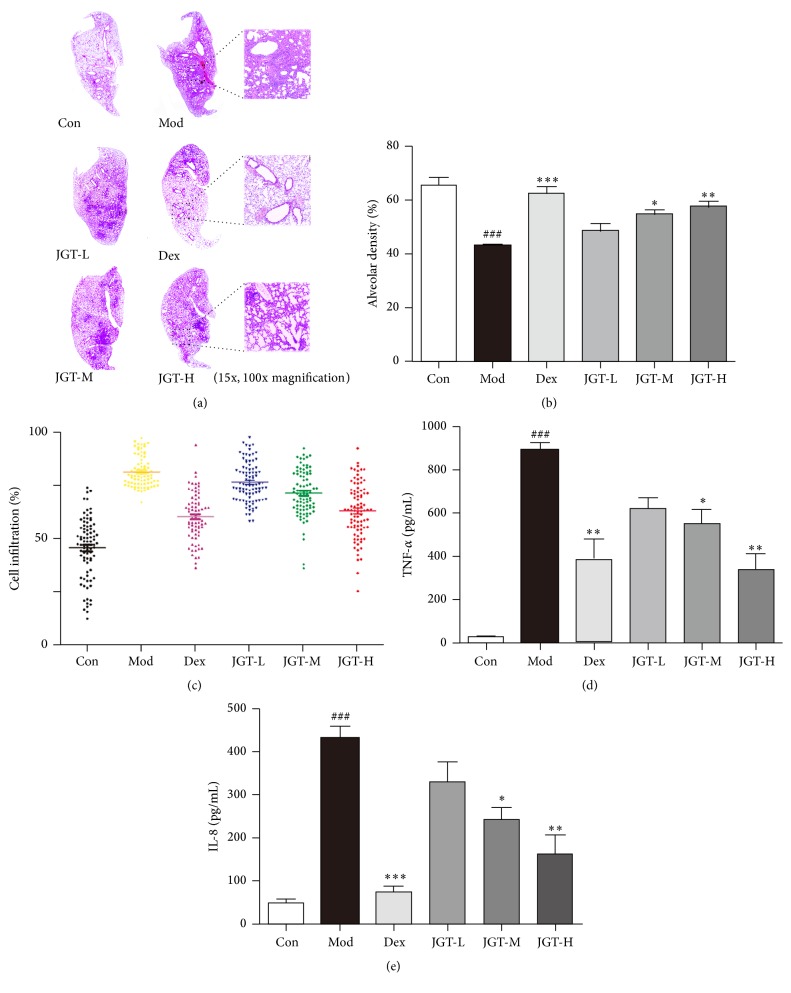
Effects of JGT prophylactic administration on acute lung inflammation induced by LPS. (a) HE staining of lung cross sections. The pictures were taken by light microscopy and partially enlarged with 10x magnification. The pathological classification of lung injury shown by the inflammatory infiltration was expressed as the alveolar density (b) and the average gray values of the images (c). Effects of JGT on (d) TNF-*α* and (e) IL-8 production in the BALF. Values are presented as the mean ± SEM. ^###^ *p* < 0.001* versus* Con group; ^*∗*^ *p* < 0.05, ^*∗∗*^ *p* < 0.01, and ^*∗∗∗*^ *p* < 0.001* versus* Mod group (*n* = 6).

To evaluate the prophylactic administration effects of JGT on ALI, we evaluated histological changes among the six groups in the LPS- or non-LPS-treated mice. The cross-sectional images of lung tissues and the local amplified images are shown in Figure 3(a). The pathological classification of lung injury was based on the alveolar density percentage (Figure 3(b)) and the infiltration of inflammatory cells was expressed as the overall average gray value (Figure 3(c)). In the Mod group, the lungs were significantly damaged with inflammatory cell infiltration, alveolar wall thickening, and interstitial edema. In contrast, significantly reduced histological injury was found in the Dex group (*p* < 0.001), with less obstruction of small airways and recruitment of inflammatory infiltrates. Although the histological damage was not improved by the low JGT dose, pretreatment with the medium and high JGT doses (1.35 g/kg or 4.05 g/kg) relieved the inflammatory cell infiltration and interstitial edema to different degrees (*p* < 0.05 and *p* < 0.01). The results indicated that JGT ameliorated the inflammation caused by LPS-induced ALI in mice.

### 3.3. Effects of JGT on Cytokine and Chemokine Production in the BALF

To evaluate the cytokine (TNF-*α*) and chemokine (IL-8) levels in the BALF, BALF was collected 24 h after LPS administration. The effects of JGT on TNF-*α* and IL-8 production were analyzed after LPS challenge by ELISA. As shown in Figures 3(d) and 3(e), the TNF-*α* and IL-8 concentrations in the BALF were significantly increased in response to LPS stimulation compared to the Con group. Dex significantly reduced TNF-*α* and IL-8 production compared to the Mod group (*p* < 0.01 and *p* < 0.001, resp.). Pretreatment with the low JGT dose (0.45 g/kg) did not prevent the release of TNF-*α* and IL-8; however, treatment with the medium and high JGT doses (1.35 g/kg or 4.05 g/kg) decreased the production of the inflammatory factors to varying degrees (*p* < 0.05 and *p* < 0.01, resp.).

### 3.4. iTRAQ Analysis and Protein Interaction Analysis

**Figure 4:**
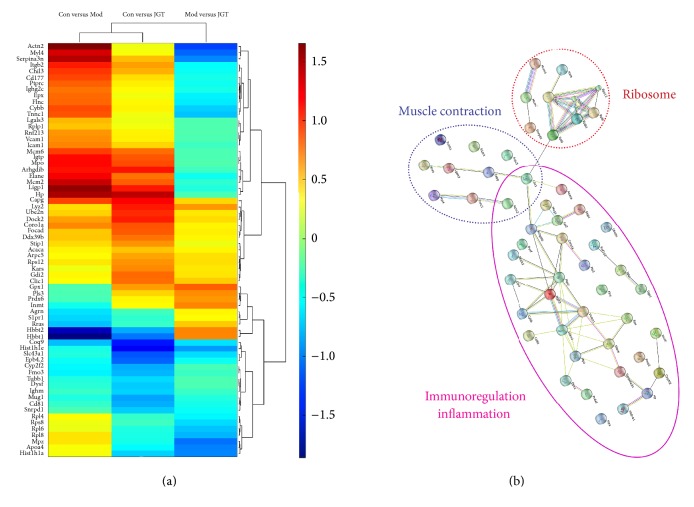
Proteomics analysis using mouse lung tissues induced by LPS. (a) Heat map of the iTRAQ differentially expressed protein analysis. (b) Protein interaction analysis by the bioinformatics tool String 9.1.

To evaluate the different proteins included in the three groups of samples, samples from the three groups were processed by cluster analysis to assess similar protein expression patterns that typically shared similar functions. The data values were standardized, the Euclidean distances between the data were calculated, and the quantitative protein and experimental conditions were evaluated for the hierarchical clustering analysis using the SIMC-P software. The heat map of the protein arrays based on hierarchical clustering is depicted in Figure 4(a). Each row in the figure represents a protein, each column represents a comparative group, and different colors represent different fold differences. Red indicates upregulated proteins and green indicates downregulated proteins. From the iTRAQ differential analysis, we obtained 67 different proteins that participated in 93 different pathways. The primary pathways were immune (B-cell receptor, T-cell receptor, and primary immunodeficiency), inflammation (MAPK, VEGF, and PI3K/Akt), cell movement (Gap junction), airway remodeling of the cytoskeleton and extracellular matrix (regulation of actin cytoskeleton and actin cytoskeleton), cell adhesion (CAMs), and signal transduction (adherens junction and focal adhesion). Some pathways had a variety of functions (i.e., the Wnt signaling pathway, which participated in the inflammatory reaction and muscle contraction). The expression profile of the JGT group was more similar to the Con group, which suggested that the overall protein expression profiles were inclined to normal levels with JGT treatment, indicating the mitigation and improvement of inflammation.

The bioinformatics tools String 9.1 and KEGG were applied to study the target protein interactions. Three functional proteomics categories were used based on the different proteins (immunoregulation and anti-inflammation, ribosome, and muscle contraction, resp.). The protein interaction analysis is shown in Figure 4(b). We found that these three protein functions were inseparable from the pharmacological actions of JGT.

### 3.5. Network and AutoDock Analysis

**Figure 5:**
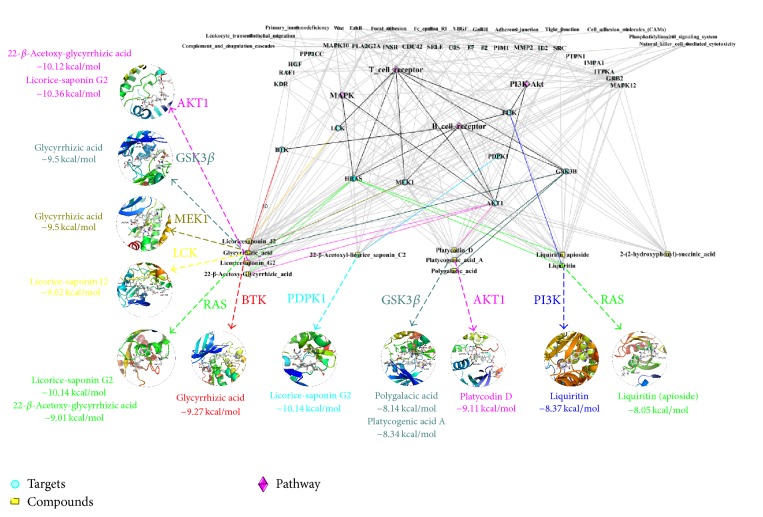
The relationship between the bioactive ingredients and predicted targets and pathways through network pharmacology and AutoDock analysis.

To study the relationship between the compounds and the predicted pathways and targets, we applied the PharmMapper database, KEGG database, and Cytoscape software to integrate the network pharmacology. Eventually, 28 predicted targets and 17 primary pathways related to the inflammatory response were found through the compound-target-pathway network (Figure 5). Among them, 8 targets (PI3K, GSK3*β*, LCK, RAS, PDPK1, AKT, MEK1, and BTK) and 4 signaling pathways (MAPK, PI3K/Akt, T-cell receptor, and B-cell receptor) were closely related to the iTRAQ protein analysis. Thus, we investigated the relationship between the bioactive compounds and the targets and pathways mentioned above. The AutoDock 4.0 software was applied to study the interaction relationships between the forecasted targets and compounds. Based on laboratory experience, −8 was regarded as the critical value. Values less than −8 were considered stable and reliable binding; otherwise, the values represented unreliable binding. The binding energy is summarized in Figure 5.

## 4. Discussion

LPS-induced ALI was characterized by the release of a variety of proinflammatory mediators. Monocyte activation leads to the release of various cytokines. These cytokines (especially TNF-*α* and IL-8) not only amplify the inflammatory cascade and cause inflammatory injury but also recruit neutrophils into the lung [15, 16]. In this study, JGT pretreatment reduced the LPS-induced increase in TNF-*α* production in a dose-dependent manner (Figure 3(d)), suggesting that JGT may be a potent anti-inflammatory compound capable of reducing neuronal injury. Consequently, cultured HEK 293 cells induced with TNF-*α* were used to build an inflammation model for the assessment of the anti-inflammatory effects of JGT* in vitro*. Eleven candidate ingredients from JGT were utilized to investigate the anti-inflammatory mechanisms. A large amount of literature has reported the anti-inflammatory effects of PG and GU but has seldom introduced the specific components that act as inflammatory inhibitors. In this study, the anti-inflammatory ingredients were screened and many were reported to have anti-inflammatory activities for the first time, such as 22-*β*-acetoxy-glycyrrhizic acid, licorice-saponin G2, 22-*β*-acetoxyl-licorice-saponin C2, and licorice-saponin J2 [17–19].

**Figure 6:**
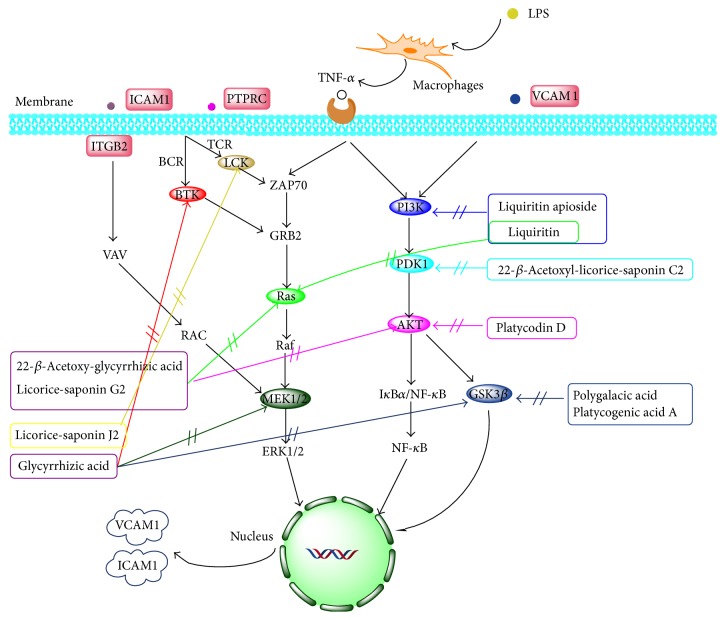
Network anti-inflammatory mechanism of JGT on LPS-induced ALI.

Based on the PharmMapper and KEGG database analyses, the anti-inflammatory ingredients primarily acted on the PI3K/Akt and ERK/MAPK signaling pathways. NF-*κ*B is a transcription factor that functions downstream of these two pathways. The network depiction of the anti-inflammatory mechanism of JGT on LPS-induced ALI is illustrated in Figure 6.

Most active compounds in PG and GU are pentacyclic triterpenoids; this type of compound inhibits IKK-mediated activation of the NF-*κ*B pathway [20]. Platycodin D was reported to modulate PI3K/Akt by inhibiting p-Akt (Ser473) [21]. Platycodin D (−9.11 kcal/mol) docked with AKT, which was in agreement with the existing docking data. Glycyrrhizic acid was reported to reduce inflammation via the PI3K/Akt/GSK3*β* pathways [22]. Our results revealed that glycyrrhizic acid (−9.51 kcal/mol) and 22-*β*-acetoxy-glycyrrhizic acid (−10.36 kcal/mol) had good affinity for GSK3*β* and AKT, respectively. Additionally, platycogenic acid A and polygalacic acid had binding energies of −8.14 and −8.34 kcal/mol for GSK3*β*, respectively. Based on these results, we deduced that glycyrrhizic acid, platycogenic acid A, and polygalacic acid jointly mediated the interaction with GSK3*β*. Licorice-saponin G2 (−10.36 kcal/mol) also bound effectively to AKT. 22-*β*-Acetoxyl-licorice-saponin C2 showed a latent affinity for the target PDPK1 in the PI3K/Akt pathway with a score of −8.82 kcal/mol. The binding energies of liquiritin for PI3K and RAS were −8.37 and −8.05 kcal/mol, respectively. PI3K is an upstream protein of AKT and GSK3*β* and RAS is an upstream protein of ERK. Liquiritin was found to enhance the phosphoactivation of ERKs, AKT, and downstream GSK3*β* [23], suggesting that PI3K might be the potential target of liquiritin. Above all, we can deduce that PG and GU comediate the PI3K/Akt pathways to inhibit the release of NF-*κ*B.

GU showed a remarkable inhibitory effect on the ERK/MAPK pathway according to the docking results and literature [24]. LCK and BTK were modulated by PTPRC via the T-cell receptor and B-cell receptor pathways, respectively [25]. LCK and BTK were primarily affected by licorice-saponin J2 (−9.02 kcal/mol) and glycyrrhizic acid (−9.72 kcal/mol); these proteins were upstream proteins of Ras in the MAPK pathway [26]. RAS was comediated by licorice-saponin G2 (−10.14 kcal/mol) and liquiritin (−8.05 kcal/mol) [27]. Furthermore, glycyrrhizic acid emerged with the potential to act on MEK1 with a score of −8.78, which was experimentally proven [28]. The above data demonstrate that GU plays the major role in inhibiting the MAPK pathway to reduce inflammation. Moreover, glycyrrhizic acid, which is the primary bioactive compound in GU, plays the dominant role in the suppression of the inflammatory reaction.

In the iTRAQ analysis, PTPRC, VCAM1, ICAM1, and ITGB2 expression was reduced. Notably, these four proteins participate in the PI3K/Akt and ERK/MAPK signaling pathways. PTPRC (also known as CD45) plays a role in the protein tyrosine phosphatase- (PTPase-) dependent signaling cascade that results in the activation of Ras and leads to a Rafl/MEK/MAPK module [29]. The upregulation of CD45 after LPS stimulation indicated that a T-cell receptor signaling pathway or a MEK/MAPK pathway participated in the regulation of the inflammatory response. VCAM1 (vascular cell adhesion molecule 1) is a member of the immunoglobulin (Ig) superfamily that mediates the adhesion of lymphocytes, monocytes, eosinophils, and basophils to the vascular endothelium [30]. ICAM1 (intercellular adhesion molecule 1) is a member of the immunoglobulin superfamily that possesses binding sites for a number of immune-associated ligands [31]. Studies have shown that MAPK/NF-*κ*B activation can produce VCAM1 and ICAM1; upregulation of VCAM1 in endothelial cells by cytokines occurs as a result of increased gene transcription (e.g., in response to TNF-*α*). Therefore, VCAM1 and ICAM1 expression can be used as criteria for inflammation. Our available data showed that LPS stimulation upregulated VCAM1 and ICAM1 expression, whereas JGT treatment reduced the expression of these two proteins. ITGB2 (integrin beta-2) belongs to the leukocyte integrin subfamily and is known to participate in cell adhesion and the cell surface-mediated signaling pathway [32]. ICAM1 binds to ITGB2 to facilitate the transmigration of leukocytes across the vascular endothelia in processes such as extravasation and the inflammatory response [33]. As a result of these binding characteristics, ICAM1 has classically been assigned the function of intercellular adhesion. Thus, the expression of ITGB2 is in accordance with ICAM1 in theory. Our present work showed a significant decrease in ITGB2 following JGT administration. These results reveal a therapeutic effect on LPS-induced ALI.

## 5. Conclusions

In summary, the present study provided experimental evidence that JGT exerted ameliorating effects on LPS-induced ALI. JGT also suppressed the expression of inflammatory mediators and inhibited the activation of NF-*κ*B. Eleven potential anti-inflammatory ingredients were found, including the derivatives of glycyrrhizic acid, licorice-saponin, liquiritin, and platycodigenin. PG and GU comediated PI3K/Akt signal pathway inhibition of NF-*κ*B, VCAM1, and ICAM1 release which primarily acted on PI3K, PDK1, AKT, and GSK3*β*. GU markedly inhibited the ERK/MAPK signaling pathways and primarily acted on LCK, Ras, and MEK. Chemomics-integrated proteomics analysis demonstrated that multiple targets of different components were related to the anti-inflammatory effects that contributed to the clinical efficacy of JGT for the treatment of ALI.

## Figures and Tables

**Table 1:** UPLC-DAD/Q-TOF-MS identification of the bioactive constituents in JGT.

Peak number	*t*/*R* (min)	Identification	Mode	MS (*m*/*z*)	Error/ppm	MS/MS	Monoisotopic mass	Composition	Source
1	4.2	2-(2-Hydroxyphenyl)-succinic acid	Neg.	209.0509	1.96	209 [M − H]^−^; 165 [M − H − CO_2_]^−^; 129 [M − H − CO_2_ − 2H_2_O]^−^; 121 [M − H − 2CO_2_]^−^	210.0528	C_10_H_10_O_5_	GU

2	7.32	Liquiritin apioside	Pos.	551.1744	−3.63	551 [M + H]^+^; 419 [M + H − api]^+^; 257 [M + H − api − glc]^+^	550.1686	C_26_H_30_O_13_	GU
			Neg.	549.1657	8.9	549 [M − H]^−^; 417 [M − H − api]^−^; 255 [M − H − api − glc]^−^			

3	7.57	Liquiritin	Pos.	419.1335	1.67	419 [M + H]^+^; 257 [M + H − glc]^+^	418.1264	C_21_H_22_O_9_	GU
			Neg.	417.1212	6.23	417 [M − H]^−^; 255 [M − H − glc]^−^			

4	8.36	Platycogenic acid A	Neg.	533.314	4.69	533 [M − H]^−^	534.3193	C_30_H_46_O_8_	PG

5	8.97	Polygalacic acid	Pos.	505.3437	8.72	505 [M + H]	504.3451	C_30_H_48_O_6_	PG

6	11.55	Licorice-saponin J2	Pos.	825.427	0.24	825 [M + H]^+^; 649 [M + H − glu]^+^; 455 [M + H − glu − Glu]^+^	824.4194	C_42_H_64_O_16_	GU
			Neg.	823.418	7.77	823 [M − H]^−^; 351 [2 × C_6_H_8_O_6_ − H]^−^; 191 [C_6_H_8_O_6_ − H]^−^			

6′	11.95	Platycodin D	Pos.	1225.5932	− 6.64	1225 [M + H]^+^	1224.5775	C_57_H_92_O_28_	PG
			Neg.	1223.5737	3.27	1223 [M − H]^−^; 681 [M − C_21_H_34_O_16_]^−^			

7	13.27	22-*β*-Acetoxy-glycyrrhizic acid	Pos.	881.4104	− 7.6	881 [M + H]^+^; 705 [M + H − glu]^+^; 511 [M + H − glu − Glu]^+^	880.4092	C_44_H_64_O_18_	GU
			Neg.	879.4095	9.10	879 [M − H]^−^; 351 [2 × C_6_H_8_O_6_ − H]^−^			

8	14.64	Licorice-saponin G2	Pos.	839.4102	4.41	839 [M + H]^+^; 663 [M + H − glu]^+^; 469 [M + H − glu − Glu]^+^	838.3987	C_42_H_62_O_17_	GU
			Neg.	837.3927	2.15	837 [M − H]^−^; 819 [M − H − H_2_O]^−^; 351 [2 × C_6_H_8_O_6_ − H]^−^			

9	15.07	22-*β*-Acetoxyl-licorice-saponin C2	Neg.	863.4142	2.43	863 [M − H]^−^; 351 [2 × C_6_H_8_O_6_ − H]^−^	864.4144	C_44_H_64_O_17_	GU

10	15.55	Glycyrrhizic acid	Pos.	823.4043	− 8.86	823 [M + H]^+^; 647 [M + H − glu]^+^; 453 [M + H − glu − Glu]^+^	822.4038	C_42_H_62_O_16_	GU
			Neg.	821.3983	2.8	821 [M − H]^−^; 351 [2 × C_6_H_8_O_6_ − H]^−^			

glc: glucose-H_2_O; Glu: glucuronide; glu: glucuronide-H_2_O; api: apiose-H_2_O.

## Abbreviations

JGT: Jie-Geng-Tang
TCM: Traditional Chinese medicine
PG: * Platycodon grandiflorum* (Jacq.) A. DC.
GU: * Glycyrrhiza uralensis* Fisch.
ALI: Acute lung injury
iTRAQ: Isobaric tags for relative and absolute quantitation
LPS: Lipopolysaccharide
Dex: Dexamethasone
HEK: Human embryonic kidney
Con: Control
Mod: Model
BALF: Bronchoalveolar lavage fluid
HE: Histopathological examination
GA: Genetic algorithm.
